# Ancient evolutionary origins of hepatitis E virus in rodents

**DOI:** 10.1073/pnas.2413665121

**Published:** 2024-12-09

**Authors:** Wendy K. Jo, Murilo Henrique Anzolini Cassiano, Edmilson Ferreira de Oliveira-Filho, Sebastian Brünink, Adiya Yansanjav, Mesele Yihune, Alyona I. Koshkina, Alexander N. Lukashev, Leonid A. Lavrenchenko, Vladimir S. Lebedev, Ayodeji Olayemi, Umaru Bangura, Mónica Salas-Rojas, Álvaro Aguilar-Setién, Elisabeth Fichet-Calvet, Jan Felix Drexler

**Affiliations:** ^a^Institute of Virology, Charité-Universitätsmedizin Berlin, Corporate Member of Freie Universität Berlin and Humboldt-Universität zu Berlin, Berlin 10117, Germany; ^b^Mammalian Ecology Laboratory, Institute of Biology, Mongolian Academy of Sciences, Ulaanbaatar 13330, Mongolia; ^c^Department of Zoological Sciences, Addis Ababa University, Addis Ababa 1176, Ethiopia; ^d^Association for the Conservation of Biodiversity of Kazakhstan, Astana 010000, Kazakhstan; ^e^Martsinovsky Institute of Medical Parasitology, Tropical and Vector Borne Diseases, Sechenov First Moscow State Medical University, Moscow 119435, Russia; ^f^Department of Mammalian Microevolution, Severtsov Institute of Ecology and Evolution, Russian Academy of Sciences, Moscow 119071, Russia; ^g^Zoological Museum, Moscow State University, Moscow 125009, Russia; ^h^Natural History Museum, Obafemi Awolowo University, Ile-Ife, Osun State 220005, Nigeria; ^i^Department of Public Health, College of Medical Sciences, Njala University, Bo 232032, Sierra Leone; ^j^Implementation Research/Zoonoses Control, Bernhard Nocht Institute for Tropical Medicine, Hamburg D-20324, Germany; ^k^Unidad de Investigación Médica en Inmunología, Hospital de Pediatría, Centro Médico Nacional “Siglo XXI”, Instituto Mexicano del Seguro Social, Mexico City 06720, Mexico; ^l^German Centre for Infection Research (DZIF), Charité-Universitätsmedizin Berlin, Berlin 10117, Germany

**Keywords:** hepatitis E virus, evolution, rodent, zoonosis, host

## Abstract

Hepatitis E virus (HEV; family *Hepeviridae*) infections cause >40,000 human deaths annually. Zoonotic infections predominantly originate from ungulates and occasionally from rats, highlighting the zoonotic potential of rodent-associated hepeviruses. We conducted host genomic data mining and uncovered two genetically divergent rodent-associated hepeviruses, and two bat-associated hepeviruses genetically related to known bat-associated strains. We thus analyzed 2,565 liver specimens from 108 rodent and shrew species sampled from globally understudied regions and hosts in Africa, Asia, and Latin America during 2011-2018 for hepeviruses by RT–PCR. We detected 63 positive field samples (2.5%, 95% CI 1.9-3.1) from 14 animal species, including two coinfections with genetically divergent strains and significant variation (*X*^2^, *P* < 0.001) in detection rates between study sites. Strain-specific qRT–PCR assays showed virus concentrations between 9.2 × 10^2^ and 3.2 × 10^9^ copies/g. We recovered 24 near-complete hepeviral genomes from rodents, shrews, and bats, all showing three partially overlapping open reading frames (ORFs), some including putative late domains that may be associated with quasi-envelopment. Rodent-derived hepeviruses grouped into five clades clustering in basal sister relationship to human- (31 to 84% distance in translated ORF1-3) and rat-associated HEV. Parsimony-based analyses and cophylogenetic reconciliations revealed that rodents were predominant sources of hepeviral cross-order host shifts. Bayesian ancestral state reconstructions substantiated a direct origin of human-associated HEV in ungulates such as swine and camelids (posterior probability 0.8), whereas the nonrecent evolutionary origins of human- and ungulate-associated HEV were projected to rodent hosts (posterior probability > 0.9). Our results elucidate the genealogy of human HEV and warrant increased surveillance and experimental risk assessments for rodent-associated hepeviruses.

Hepatitis E virus (HEV; family *Hepeviridae*) annually causes 20 million infections and 44,000 human deaths worldwide (1) despite regional availability of a licensed vaccine (2). Of the eight HEV genotypes (genus *Paslahepevirus*), humans are the reservoir hosts of genotypes 1 and 2, whereas the other genotypes have a nonhuman animal reservoir (swine, rabbit, or camelid) (3). Genotypes 3 and 4 are zoonotic and account for most cases in industrialized countries. Rodents, the most speciose mammals, harbor major zoonotic pathogens (4, 5), including rat HEV (RHEV; genus *Rocahepevirus*) sporadically found in humans (6). Here, we characterize divergent hepeviruses from combined data mining/fieldwork and show that rodents are major drivers of the hepeviral genealogy.

## Results and Discussion

**Fig. 1.**
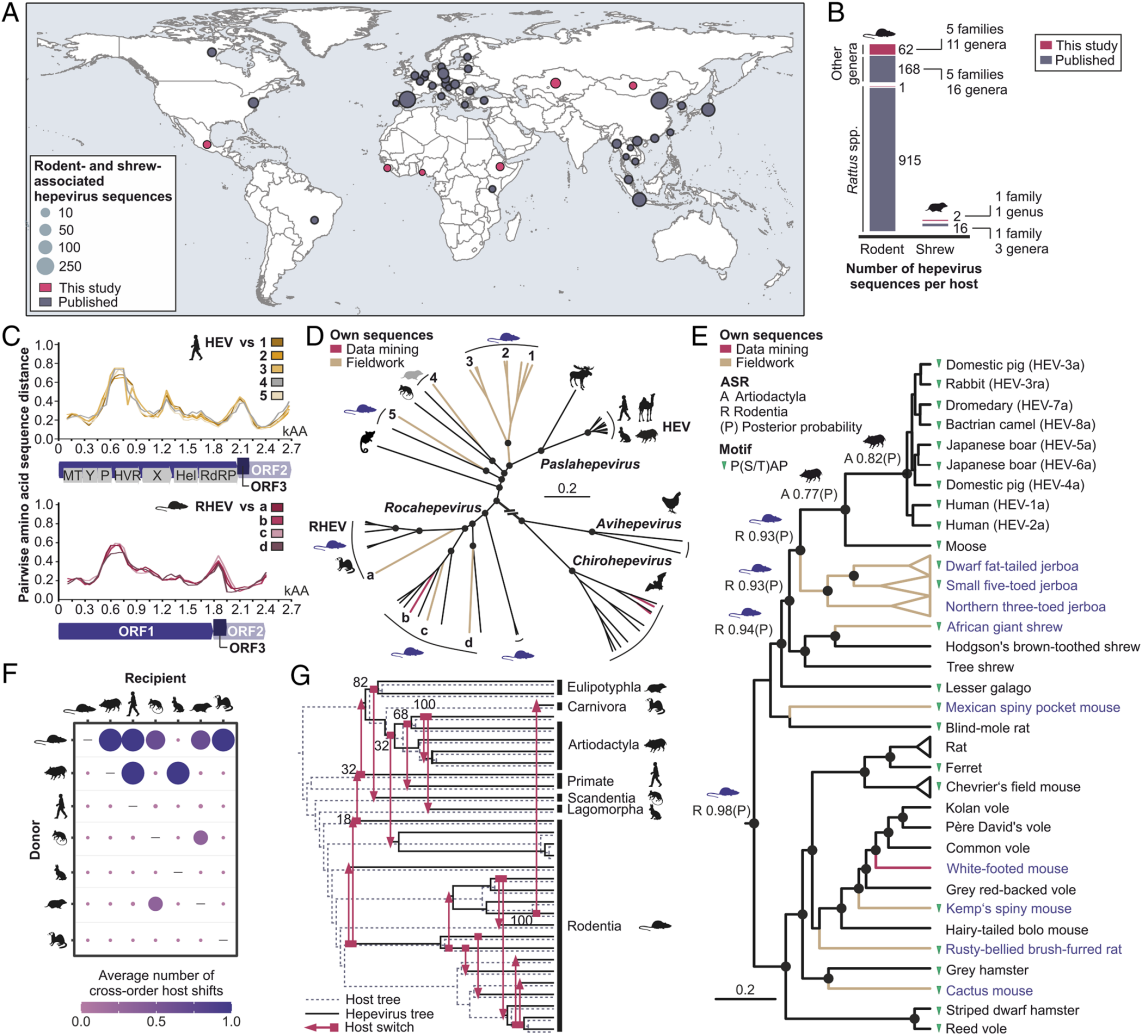
Hepevirus epidemiology and evolution. (*A*) Geographic representation of hepeviruses identified in rodents and shrews. (*B*) Hepevirus sequence counts per host. (*C*) Pairwise amino acid sequence distance plots (SSE v1.4). HEV L08816, RHEV MZ868954. Numbers, sequences clustering with paslahepeviruses; letters, sequences within rocahepeviruses (panel *D*). (*D*) *Orthohepevirinae* Bayesian tree. (*E*) Bayesian ancestral state reconstruction (ASR), *Pasla*-/*Rocahepevirus* genera. Host order support with highest posterior probability at major nodes. Triangles, late domain motifs. Taxa with branch lengths <0.1 and the same host species were collapsed. Panels (*D* and *E*) Circles, posterior probability support of grouping ≥0.9 (Beast V1.10.4). Scale bar, genetic distance. (*F*) Parsimony-based average number of cross-order host shifts from donor to recipient hosts (Mesquite 3.81). (*G*) Event-based cophylogeny (eMPRess v1). Numbers, event frequency.

Recently, 2 divergent hepeviruses were uncovered in host genomic data from a nonhuman primate (lesser galago; *Galago senegalensis*) and rodents (blind-mole rats; *Nannospalax galili*) (7). To comparatively investigate divergent hepeviruses, we analyzed 7.67 million (M) sequencing runs (www.ncbi.nlm.nih.gov/sra/), including 3.52 M runs from mammals, of which 1.74 M were from primates, 1.65 M from rodents, 102,427 from artiodactyls, 3,096 from bats, and 395 from shrews. Within the 137 hepeviruses identified from publicly available data (Dataset S1), only 4 were genetically divergent from known hepeviruses based on the phylogenetic distance segregating HEV genotypes (≥0.03 branch length) in a neighbor-joining tree relying on concatenated open reading frames (ORF) 1 and 2 (SI Appendix). The sequences identified included two genetically divergent rodent-associated hepeviruses in deermice, and two bat-associated hepeviruses in horseshoe bats genetically related to known horseshoe bat-associated strains. Despite numerous published studies, hepeviruses from rodents and often sympatric shrews have only been described from a small number of countries globally (Fig. 1*A* and Dataset S2). Most of the sequences (84.5%; 915/1,083) belonged to *Rattus* spp. and only 15.5% (168/1,083) have been identified in other rodent hosts (Fig. 1*B* and Dataset S2). We thus tested 2,565 animals belonging to 108 rodent and shrew species sampled during 2011-2018 from largely unexplored regions for HEV-related viruses, including Sierra Leone, Guinea, Nigeria, Ethiopia, Mongolia, Kazakhstan, and Mexico (Dataset S3). We found 63 positives (2.5%, 95% CI 1.9-3.1) by screening liver samples using broadly reactive nested RT–PCR, of which 61 were from 12 rodent species belonging to 5 families and 2 were from 2 shrew species from the same family (Dataset S3). The detection rates varied significantly across countries (*X*², *P* < 0.001; 0.3 to 25.4%, Dataset S4) and host species (0.7 to 100%, Dataset S5). Rodent livers had high viral loads with a median value of 7.4 × 10^6^ (range, 9.2 × 10^2^ to 3.2 × 10^9^) copies/g (Dataset S3). Combining data mining and fieldwork, we retrieved 24 near-complete hepevirus genomes (Dataset S6). All genomes had a typical hepevirus organization containing ORF1, ORF2, and ORF3 and typical functional domains and motifs (Dataset S7). A putative ORF4 was not consistently found in rodent-associated hepeviruses (SI Appendix). All rodent-/shrew-associated hepeviruses were highly divergent from paslahepeviruses and rocaviruses, showing pairwise amino acid sequence distances of 38 to 50% and 32 to 53% in ORF1, 31 to 44% and 23 to 46% in ORF2, and 61 to 84% and 64 to 88% in ORF3, respectively (Dataset S8). Sequence distances were highest around the ORF1 hypervariable region and the ORF3 (Fig. 1*C*), potentially due to host adaptation or recombination events. ORF3 of rodent-associated hepeviruses and birds, but not bats (Dataset S9), showed canonical late domain motifs (P[S/T]AP) associated with quasi-envelopment in human HEV (8). Whether the ability to form quasi-enveloped virions preceded the formation of human-associated HEV is an intriguing question, but the functionality of those putative late domains remains to be investigated.

Two dwarf fat-tailed jerboas (*Pygeretmus pumilio*) were coinfected with two hepeviruses showing >20% translated amino acid sequence divergence from each other, highlighting intense circulation of divergent hepeviruses in these rodents. Following formal testing and exclusion of potentially recombinant genomic regions (SI Appendix), Bayesian phylogenetic analyses of a dataset encompassing approximately 1,400 amino acids derived from ORF1/2 revealed that rodent hepeviruses were not monophyletic (Fig. 1*D*). Together with the RHEV clade, rodent-associated hepeviruses formed five clades clustering in basal sister relationship to paslahepeviruses and rocahepeviruses. Rodents hosted a large diversity of hepeviruses in contrast to bat- and bird-associated hepeviruses, which formed monophyletic clades that grouped distantly to human-associated hepeviruses (Fig. 1*D*), which may suggest a limited role of those animals during the genealogy of human-associated HEV, as previously suggested (1).

Ancestral state reconstruction (ASR) in a Bayesian framework substantiated that human-associated HEV likely had a recent origin in even-toed ungulates such as swine and camelids (order Artiodactyla, posterior probability 0.8, Fig. 1*E*), previously associated with the domestication of pigs approximately 6,000 y ago (9). ASR also yielded decisive evidence for the nonrecent origin of human-associated HEV in small mammals (rodents, shrews, tree shrews; log_10_ Bayes factor 2.6, Dataset S10), and particularly in rodents (posterior probability > 0.9, Fig. 1*E*). Parsimony-based ASR showed that host switches predominantly originated from rodents (5 recipient orders; mean number of host switches, 0.7) compared to those from ungulates, tree shrews, and shrews (1 to 2 recipient orders; Fig. 1*F*). Event-based reconciliations of hepevirus and host phylogenies reconstructed 17 host shifts, again predominantly involving rodents (n = 11; 65%) (Fig. 1*G*). Notably, some shrew hepeviruses identified in this and previous studies (10, 11) grouped apically in rodent-associated hepevirus clades, suggesting viral cross-order host shifts between sympatric animals. Distance-based cophylogenetic reconstructions corroborated an overall long-term evolutionary association between hepeviruses and their rodent and ungulate hosts (*P* < 0.001) (Dataset S11).

Limitations of our study include nonsystematic sampling across host diversity, space, and time, testing by RT-PCR which may miss divergent hepeviruses, and uneven representation of host genomic data in public databases. However, detection of highly divergent hepeviruses, our large sample, and exhaustive data mining enabled robust evolutionary reconstructions.

RHEV models exist (12), whereas for human-associated HEV, establishment of a tractable animal model has proven challenging (13). However, immunocompetent gerbils (a murine rodent) are susceptible to both zoonotic and nonzoonotic HEV genotypes (14, 15), which is consistent with putative rodent ancestry of paslahepeviruses. Only few genetic changes may be required for efficient HEV-1 infection of gerbils. Those adaptive mutations (15) were not consistently present in rodent-associated hepeviruses, some even occurring in human-associated genotypes. Divergent rodent-associated hepeviruses and rodent hosts may thus prove beneficial in optimizing HEV infection models. Our findings thus warrant increased surveillance and experimental risk assessments for rodent-associated hepeviruses.

## Methods

Hepeviruses were identified by mining of publicly available genomic data via Serratus and by testing field samples via RT-PCR- and Illumina-based methods. Near-complete genomes were retrieved by de novo and reference assemblies. Recombination analyses (RDP5) and Bayesian phylogenies (Beast) of *Orthohepevirinae* were conducted. Macroevolutionary analyses for pasla- and rocahepeviruses involved parsimony-based (Mesquite) and Bayesian (Beast, BayesTraits) ancestral state reconstructions, as well as distance- (ParaFit) and event-based (eMPRess) cophylogenetic reconciliations. Permits and methods are detailed in SI Appendix.

## Supplementary Material

Appendix 01 (PDF)

Dataset S01 (XLSX)

Dataset S02 (XLSX)

Dataset S03 (XLSX)

Dataset S04 (XLSX)

Dataset S05 (XLSX)

Dataset S06 (XLSX)

Dataset S07 (XLSX)

Dataset S08 (XLSX)

Dataset S09 (XLSX)

Dataset S10 (XLSX)

Dataset S11 (XLSX)

Dataset S12 (XLSX)

## Data Availability

Sequence data have been deposited in GenBank and SRA under the project PRJNA1137858 (16), with accession numbers PQ489611-PQ489675 (17). Other study data are included in the article and/or supporting information. Access to sample material is restricted due to Material Transfer Agreements and the limited availability of field specimens. Previously published data were utilized for this work; specifically rodent hepevirus sequences acquired from the NCBI Virus database up until June 25, 2024. Additionally, taxonomic information on the mammals in the datasets was gathered using version 1.10 of the Mammal Diversity Database (DOI: 10.5281/zenodo.7394529) (18) from the American Society of Mammalogists.

## References

[1] A. Rasche, A. L. Sander, V. M. Corman, J. F. Drexler, Evolutionary biology of human hepatitis viruses. J. Hepatol. **70**, 501–520 (2019). 30472320 10.1016/j.jhep.2018.11.010 PMC7114834

[2] S. Huang , Long-term efficacy of a recombinant hepatitis E vaccine in adults: 10-year results from a randomised, double-blind, placebo-controlled, phase 3 trial. Lancet **403**, 813–823 (2024). 38387470 10.1016/S0140-6736(23)02234-1

[3] M. A. Purdy , ICTV virus taxonomy profile: Hepeviridae 2022. J. Gen. Virol. **103**, 001778 (2022). 10.1099/jgv.0.001778 PMC12642825 36170152

[4] N. Mollentze, D. G. Streicker, Viral zoonotic risk is homogenous among taxonomic orders of mammalian and avian reservoir hosts. Proc. Natl. Acad. Sci. U.S.A. **117**, 9423–9430 (2020). 32284401 10.1073/pnas.1919176117 PMC7196766

[5] B. A. Han, J. P. Schmidt, S. E. Bowden, J. M. Drake, Rodent reservoirs of future zoonotic diseases. Proc. Natl. Acad. Sci. U.S.A. **112**, 7039–7044 (2015). 26038558 10.1073/pnas.1501598112 PMC4460448

[6] A. Rivero-Juarez , Orthohepevirus C infection as an emerging cause of acute hepatitis in Spain: First report in Europe. J. Hepatol. **77**, 326–331 (2022). 35167911 10.1016/j.jhep.2022.01.028

[7] J. Kawasaki, S. Kojima, K. Tomonaga, M. Horie, Hidden viral sequences in public sequencing data and warning for future emerging diseases. mBio **12**, e0163821 (2021). 34399612 10.1128/mBio.01638-21 PMC8406186

[8] A. Das , Cell entry and release of quasi-enveloped human hepatitis viruses. Nat. Rev. Microbiol. **21**, 573–589 (2023). 37185947 10.1038/s41579-023-00889-z PMC10127183

[9] S. Baha , Comprehensive analysis of genetic and evolutionary features of the hepatitis E virus. BMC Genomics **20**, 790 (2019). 31664890 10.1186/s12864-019-6100-8 PMC6820953

[10] W. He , The prevalence and genomic characteristics of hepatitis E virus in murine rodents and house shrews from several regions in China. BMC Vet. Res. **14**, 414 (2018). 30577796 10.1186/s12917-018-1746-z PMC6303920

[11] Q. Ding , Prevalence and molecular characterization of hepatitis E virus (HEV) from wild rodents in Hubei Province, China. Infect. Genet. Evol. **121**, 105602 (2024). 38734397 10.1016/j.meegid.2024.105602

[12] X. Zhang , Establishment of a robust rat hepatitis E virus fecal-oral infection model and validation for antiviral studies. Antiviral. Res. **216**, 105670 (2023). 37451630 10.1016/j.antiviral.2023.105670

[13] I. M. Sayed, A. A. Elkhawaga, M. A. El-Mokhtar, In vivo models for studying Hepatitis E virus infection; Updates and applications. Virus Res. **274**, 197765 (2019). 31563457 10.1016/j.virusres.2019.197765

[14] S. Subramaniam , Distinct disease features of acute and persistent genotype 3 hepatitis E virus infection in immunocompetent and immunosuppressed Mongolian gerbils. PLoS Pathog. **19**, e1011664 (2023). 37703304 10.1371/journal.ppat.1011664 PMC10519604

[15] T. Liu , An immunocompetent Mongolian gerbil model for hepatitis E virus genotype 1 infection. Gastroenterology **167**, 750–763.e10 (2024). 38582270 10.1053/j.gastro.2024.03.038

[16] M. Anzolini Cassiano , Divergent hepeviruses. NCBI. https://www.ncbi.nlm.nih.gov/bioproject/PRJNA1137858. Deposited 19 July 2024.

[17] W. K. Jo , GenBank accession no. PQ489631.1. NCBI. https://www.ncbi.nlm.nih.gov/nuccore/2845860111. Deposited 18 October 2024.

[18] N. Upham , Mammal Diversity Database (version 1.10). Zenodo. 10.5281/zenodo.7394529. Accessed 25 June 2024.

