# Supplementary material for: Ancient evolutionary origins of hepatitis E virus in rodents
Source: Proc Natl Acad Sci U S A. 2024 Dec 9;121(51):e2413665121. doi: 10.1073/pnas.2413665121 (PMC11665899; doi:10.1073/pnas.2413665121)
Supplement: Supplementary file 1 — Appendix 01 (PDF) [file pnas.2413665121.sapp.pdf]

PNAS

Supporting Information for
Ancient evolutionary origins of hepatitis E virus in rodents

Wendy K. Jo[a], Murilo Henrique Anzolini Cassiano[a], Edmilson Ferreira de Oliveira-Filho[a], Sebastian Brünink[a], Adiya Yansanjav[b], Mesele Yihune[c], Alyona I. Koshkina[d], Alexander N Lukashev[e], Leonid A. Lavrenchenko[f], Vladimir S. Lebedev[g], Ayodeji Olayemi[h], Umaru Bangura[i,j], Mónica Salas-Rojas[k], Álvaro Aguilar-Setién[k], Elisabeth Fichet-Calvet[j], Jan Felix Drexler[a]

[a] Charité-Universitätsmedizin Berlin, corporate member of Freie Universität Berlin and Humboldt-Universität zu Berlin, Institute of Virology, Berlin 10117, Germany
[b] Institute of Biology, Mongolian Academy of Sciences, Ulaanbaatar, Mongolia
[c] Department of Zoological Sciences, Addis Ababa University, Addis Ababa 1176, Ethiopia
[d] Association for the Conservation of Biodiversity of Kazakhstan, Astana 010000, Kazakhstan
[e] Martsinovsky Institute of Medical Parasitology, Tropical and Vector Borne Diseases, Sechenov First Moscow State Medical University, Moscow 119435, Russia
[f] Department of Mammalian Microevolution, A. N. Severtsov Institute of Ecology and Evolution, Russian Academy of Sciences, Moscow 119071, Russia
[g] Zoological Museum, Moscow State University, Moscow 125009, Russia
[h] Natural History Museum, Obafemi Awolowo University, Ile-Ife, Osun State 220005, Nigeria
[i] College of Medical Sciences, Njala University, Bo, Sierra Leone
[j] Department of Virology, Bernhard Nocht Institute for Tropical Medicine, Hamburg D-20324, Germany
[k] Unidad de Investigación Médica en Inmunología, Hospital de Pediatría, Centro Médico Nacional Siglo XXI, Instituto Mexicano del Seguro Social, Mexico City, Mexico.

Jan Felix Drexler
Email: felix.drexler@charite.de

This PDF file includes:

Supporting extended methods
SI References

Other supporting materials for this manuscript include the following:

Datasets S1 to S12

Extended Methods

Animal Sampling

Animals were sampled at different sites in Mexico, Kazakhstan, Mongolia, Ethiopia, Guinea, Sierra Leone, and Nigeria between 2011 and 2018 (***Dataset S3***). For all sampling sites, the study protocols, including capture, sampling, and testing of animals, were approved by the responsible animal ethics committees. The animals were identified by trained field biologists. The animals were anesthetized and euthanized by trained veterinarians. The euthanasia of the rodents followed the AVMA Guidelines for the Euthanasia of Animals. All efforts were made to minimize suffering of the animals. Surgical procedures were performed under sodium pentobarbital/ketamine anesthesia. Procedures were performed according to national and European legislation, namely the EU Council directive 86/609/EEC for the protection of animals. Trapping of the animals was performed as described previously (1-4). Permits for sampling and export were obtained from the local authority in each country. Rodent sampling in Mexico was licensed by Secretaría del Medio Ambiente del Gobierno de México (SEMARNAT) under permit number SGPA/DGVS/08283/12. Sampling in Ethiopia was granted by the Ethiopian Wildlife Conservation Authority (EWCA, No. 7731/336/05). Sampling in Kazakhstan was performed under cooperation agreement with the Kazakhstan Association for Biodiversity Conservation. Sampling in Mongolia was performed under cooperation agreement with Mongolian State University. Ethics approval was received from the ethics committee of the Ministry of Health and Sanitation of the Government of Sierra Leone. In Nigeria, permission to trap rodents by the Osun State Ministry of Environment and the Gwer West Local Government Area, Benue State. In Guinea, permission to trap rodents was obtained from the National Ethics Committee (2003/PFHG/05/GUI and 12/CNERS/12).

Virus detection

Frozen liver samples were homogenized with a bead-based tissue lyser, and RNA was extracted using MagNA Pure 96 viral nucleic acid extraction kit (Roche Molecular Systems, USA). Samples were screened for hepeviruses using broadly reactive hemi-nested RT–PCR as described previously (5). Positive bands were Sanger sequenced (Microsynth Seqlab). SYBR Green qPCR was performed on all positive samples. We performed high-throughput sequencing of only samples representative of a cluster as assessed by Maximum-likelihood phylogeny of screening fragments (288 nucleotides) reconstructed using IQ-TREE v2.3.4 (6) with 1,000 SH-aLRT and ultrafast bootstrap replicates (7, 8) for statistical support of grouping. RNA libraries from positive samples were prepared according to the KAPA HyperPrep manufacturer's protocol (Roche) and sequenced on a NextSeq 550 system (150 cycles paired-end).

Genome retrieval and genome characterization

Raw reads were quality trimmed, and the remaining reads were aligned to a hepevirus protein reference database (Ensemble) using DIAMOND (9). Aligned reads were extracted and *de novo* assembled using Geneious assembler within Geneious v11.1.5 (https://www.geneious.com). A reference-based assembly was then performed with the newly generated hepevirus contigs using –very-sensitive mode in Bowtie 2. The final consensus was created using Geneious, filtering matches for mapping quality of at least 20 and manually inspecting the mapped reads. Gaps were filled by sequences obtained by Sanger sequencing products of specific PCRs designed to target gap regions for each genome. Open reading frames (ORFs) were predicted using Geneious. In addition to the typical three ORFs in hepeviruses, we searched for putative ORF4. ORF4 has been reported to occur in both HEV-1 and RHEV, albeit at different positions. In HEV-1 (10), ORF4 lays in the middle of ORF1 with a size of <500nt whereas in RHEV (11), ORF4 is located at the N-terminal of ORF1 with a size of >500nt. In 10/24 retrieved genome sequences, we identified an

ORF located at the N-terminal of ORF1 with sizes varying from 219-672bp and low homology of 57-62% identity to RHEV. We found no ORF at a homologous sequence region to HEV-1. Hence, a putative ORF4 was not consistently found in the rodent-associated hepeviruses from this study.

Data mining

We searched for unidentified divergent hepeviruses by using the web tools Serratus Explorer and palmID: analysis suite based on the viral-RdRP palmprint barcoding sub-sequence (https://serratus.io/toolkit). Both tools rely on data mining of the SRA sequencing libraries. For the identification of orthohepeviruses, we used a cutoff value of ≥67% identity to blind mole-rat hepevirus used as query sequence (GenBank accession BR001719.1) for the hepevirus polymerase barcode sequences retrieved from palmID as sequences below that threshold belonged mostly to fish hosts (piscihepeviruses), freshwater and soil metagenomes (***Dataset S1***). The SRA sequences belonging to orthohepeviruses were downloaded and *de novo* assembly was performed. Contigs were BLASTXed against the NCBI non-redundant database. Hepeviral contigs were used as the reference to perform reference-based assemblies using –very-sensitive mode in Bowtie 2. Final consensus genome sequences were created from reads with at least a mapping quality of 20. Near-complete hepeviral genomes identified in the SRA sequences SRR5130449 (12), SRR8587280 (13), and SRR12432009 (14) were used for downstream analyses.

Dataset

The following search terms were used in the NCBI Virus database to acquire all published rodent hepevirus sequences until 25/06/2024: "hepatitis E virus", "hepeviridae", "hepeviridae sp.", "orthohepevirus", "paslahepevirus balayani", "rat hepatitis E virus", "rocahepevirus eothenomi", "rocahepevirus ratti", "rocahepevirus", "rocahepevirus sp.", "rodent hepevirus", and unclassified hepeviridae". Primate, artiodactyl and carnivore hosts were removed. A dataset of complete or almost complete genomes of all orthohepevirus species that were referred in ICTV (15), and divergent hepevirus sequences were retrieved from GenBank via Geneious until 03/01/2024. The open reading frames (ORF)-1 and ORF2 were translated, concatenated and aligned using the MAFFT plugin with the iterative refinement algorithm G-INS-I implemented within Geneious. Sequences with branch lengths ≥0.03 compared to other sequences according to the neighbor-joining method in Mega11 (16) were kept, resulting in a final dataset of 53 unique sequences, including the sequences obtained by data mining and fieldwork from this study (***Dataset S12***). A branch length ≥0.03 was selected based on the distance between different HEV genotypes using this dataset and tree construction method. The different datasets used for the analyses are provided in the dataset files. The mammal diversity database v1.10 (10.5281/zenodo.7394529) of the American Society of Mammalogists was used to gather taxonomic information on the mammals in the datasets.

Evolutionary analyses

Sequences were aligned with MAFFT (17) plugin with the iterative refinement algorithm G-INS-i implemented within Geneious. Sequence distances were calculated and visualized with the Simple Sequence Editor (SSE) v1.4 platform using a sliding window of 600 and step size of 150 amino acids. The permutation test implemented in RDP5 (18) was used to test the clustering of breakpoint positions. The potential hot spots are identified as window coordinates where the breakpoints counts are higher or lower than in >99% of windows at the identical location in the 1,000 permuted breakpoint density plots. For the analyses, the sequence KY432903 was omitted due to frameshifts and stop codons, and we corrected a frameshift in KX589065 at nucleotide positions 257-297. None of the genome regions in our dataset contained obvious hot-spots. However, manual inspection of the alignment, recombination analyses using RDP5 and Robinson-Foulds distances

suggested multiple acquisitions (external recombination events) in genome regions between methyltransferase and RdRP. Therefore, for the final dataset, we used conserved regions that also overlapped with those indicated by ICTV for species demarcation (15) encompassing ORF1 codon positions 1−450 (methyltransferase), ORF1 codon positions 971–1692 (RNA-directed RNA polymerase) and ORF2 codon positions 121−473 (capsid protein not including the region encoded by the overlapping ORF3) in reference to HEV-1a (GenBank accession no. L08816). A concatemer of these conserved fragments was used as a complete genome representative for further macroevolutionary analysis.

Bayesian phylogenies were generated using the BEAST package V1.10.4 (19), with the substitution model LG+F+I+G4, an uncorrelated relaxed clock with lognormal distribution, and as tree prior the Yule speciation process (20). The analyses were run for 20 million generations with 10% burn-in, sampling every 1000 steps. A run was considered to have reached convergence when the effective sample sizes of all parameters were >200. Convergence was assessed in Tracer v.1.7.1 (https://beast.community/tracer). The model selection was based on the best-fit model for the concatenated ORF1 and ORF2 of members of the subfamily *Orthohepevirinae* generated by ModelFinder (21) implemented in IQ-TREE.

Ancestral state reconstructions (ASR) using the parsimony method were performed in Mesquite V3.81 as described previously (1). Average number of cross-order host shifts was based on 15k posterior trees. ASR in a Bayesian framework was generated using BEAST package V1.10.4 as described above. We used BayesTraits V4.0.1 (22) to assess the likelihoods of small mammal and non-small mammal hosts at the initial stage within a Bayesian framework. The analysis employed scaled trees to 0.1 and all priors following an exponential distribution with a lambda value of 10. We conducted 5 million iterations, and estimated the marginal likelihood using the stepping stones approach, utilizing a sampler of 100 stones and running each stone for 1,000 iterations. Distance-based cophylogeny was tested in 1,000 randomly selected parasite trees using ParaFit (23) in R (V3.4.1) through the RStudio environment (V4.0.3), with the packages APE (V4.1) and Vegan (V2.6-2), with 1,000 random permutations of virus–host associations included to test for statistical significance. Event-based cophylogenetic analyses were performed using eMPRess v1, a systematic cophylogeny reconciliation tool (24). The event costs were fixed to 0 for speciation, 1 for sorting, 1 for duplication and 2 for host switch. Avian and bat hepevirus sequences were removed for ASR and cophylogenetic analyses due to their genetic distance to paslahepeviruses. To exclude sampling biases in particular species, only one hepevirus sequence was included per host species. Host cytochrome B sequences were obtained from GenBank and used for reconstruction of phylogenetic relationships according to the mammalian species tree (25) as described previously (26). For the Bayesian phylogeny involving all orthohepeviruses, the cutthroat trout virus (GenBank accession no. HQ731075) was used as outgroup. For ASR analyses, a bat hepevirus (GenBank accession no. MT210622) was used as outgroup.

References

1. J. F. Drexler *et al.*, Bats host major mammalian paramyxoviruses. *Nat Commun* **3**, 796 (2012).
2. J. F. Drexler *et al.*, Evidence for novel hepaciviruses in rodents. *PLoS Pathog* **9**, e1003438 (2013).
3. J. Bonwitt *et al.*, At Home with Mastomys and Rattus: Human-Rodent Interactions and Potential for Primary Transmission of Lassa Virus in Domestic Spaces. *Am J Trop Med Hyg* **96**, 935-943 (2017).
4. K. K. Yadav *et al.*, Rat hepatitis E virus (HEV) cross-species infection and transmission in pigs. *PNAS Nexus* 10.1093/pnasnexus/pgae259 (2024).
5. J. F. Drexler *et al.*, Bats worldwide carry hepatitis E virus-related viruses that form a putative novel genus within the family Hepeviridae. *J Virol* **86**, 9134-9147 (2012).
6. L. T. Nguyen, H. A. Schmidt, A. von Haeseler, B. Q. Minh, IQ-TREE: a fast and effective stochastic algorithm for estimating maximum-likelihood phylogenies. *Mol Biol Evol* **32**, 268-274 (2015).
7. D. T. Hoang, O. Chernomor, A. von Haeseler, B. Q. Minh, L. S. Vinh, UFBoot2: Improving the Ultrafast Bootstrap Approximation. *Mol Biol Evol* **35**, 518-522 (2018).
8. M. Anisimova, O. Gascuel, Approximate likelihood-ratio test for branches: A fast, accurate, and powerful alternative. *Syst Biol* **55**, 539-552 (2006).
9. B. Buchfink, K. Reuter, H. G. Drost, Sensitive protein alignments at tree-of-life scale using DIAMOND. *Nat Methods* **18**, 366-368 (2021).
10. V. P. Nair *et al.*, Endoplasmic Reticulum Stress Induced Synthesis of a Novel Viral Factor Mediates Efficient Replication of Genotype-1 Hepatitis E Virus. *PLoS Pathog* **12**, e1005521 (2016).
11. Z. Shafat, A. Ahmed, M. K. Parvez, A. Islam, S. Parveen, Intrinsically disordered regions in the rodent hepevirus proteome. *Bioinformation* **18**, 111-118 (2022).
12. University of California. RNAseq of Peromyscus: liver. NCBI. https://www.ncbi.nlm.nih.gov/sra/SRR5130449. Accessed 12 February 2024.
13. Texas Tech University. RNA-Seq of Peromyscus maniculatus: adult male liver. NCBI. https://www.ncbi.nlm.nih.gov/sra/SRR8587280. Accessed 12 February 2024.
14. Shandong First Medical University & Shandong Academy of Medical Sciences. Metatranscriptomic sequencing of Rhinolophus malayanus bats: feces samples. NCBI. https://www.ncbi.nlm.nih.gov/sra/SRR12432009. Accessed 12 February 2024.
15. M. A. Purdy *et al.*, ICTV Virus Taxonomy Profile: Hepeviridae 2022. *J Gen Virol* **103** (2022).
16. K. Tamura, G. Stecher, S. Kumar, MEGA11: Molecular Evolutionary Genetics Analysis Version 11. *Mol Biol Evol* **38**, 3022-3027 (2021).
17. K. Katoh, D. M. Standley, MAFFT multiple sequence alignment software version 7: improvements in performance and usability. *Mol Biol Evol* **30**, 772-780 (2013).
18. D. P. Martin *et al.*, RDP5: a computer program for analyzing recombination in, and removing signals of recombination from, nucleotide sequence datasets. *Virus Evol* **7**, veaa087 (2021).
19. M. A. Suchard *et al.*, Bayesian phylogenetic and phylodynamic data integration using BEAST 1.10. *Virus Evol* **4**, vey016 (2018).
20. W. K. Jo *et al.*, Natural co-infection of divergent hepatitis B and C virus homologues in carnivores. *Transbound Emerg Dis* **69**, 195-203 (2022).
21. S. Kalyaanamoorthy, B. Q. Minh, T. K. F. Wong, A. von Haeseler, L. S. Jermiin, ModelFinder: fast model selection for accurate phylogenetic estimates. *Nat Methods* **14**, 587-589 (2017).
22. A. Meade, M. Pagel, Ancestral State Reconstruction Using BayesTraits. *Methods Mol Biol* **2569**, 255-266 (2022).
23. P. Legendre, Y. Desdevises, E. Bazin, A statistical test for host-parasite coevolution. *Syst Biol* **51**, 217-234 (2002).
24. S. Santichaivekin *et al.*, eMPRess: a systematic cophylogeny reconciliation tool. *Bioinformatics* **37**, 2481-2482 (2021).
25. N. M. Foley, M. S. Springer, E. C. Teeling, Mammal madness: is the mammal tree of life not yet resolved? *Philos Trans R Soc Lond B Biol Sci* **371** (2016).
26. I. de Oliveira Carneiro *et al.*, A Novel Marsupial Hepatitis A Virus Corroborates Complex Evolutionary Patterns Shaping the Genus Hepatovirus. *J Virol* **92** (2018).
